# Safety and efficacy of asciminib treatment in chronic myeloid leukemia patients in real-life clinical practice

**DOI:** 10.1038/s41408-021-00420-8

**Published:** 2021-02-09

**Authors:** Valentín Garcia-Gutiérrez, Alejandro Luna, Juan M. Alonso-Dominguez, Natalia Estrada, Concepcion Boque, Blanca Xicoy, Pilar Giraldo, Anna Angona, Alberto Alvarez-Larrán, Fermin Sanchez-Guijo, María José Ramírez, Elvira Mora, Patricia Vélez, Ana Rosell, Mercedes Colorado Araujo, Beatriz Cuevas, Miguel Sagüés, Montserrat Cortes, Manuel Perez Encinas, Luis Felipe Casado Montero, Melania Moreno Vega, Luis Serrano, Valle Gomez, Carmen Garcia-Hernandez, Sunil Lakhwani, Antonio Paz Coll, Raquel de Paz, Sara Suarez-Varela, Andrés Fernandez-Ruiz, Raul Perez Lopez, Almudena Ortiz-Fernández, Antonio Jiménez-Velasco, Juan Luis Steegmann-Olmedillas, Juan Carlos Hernández-Boluda

**Affiliations:** 1 Hematology, Hospital Universitario Ramón y Cajal. IRYCIS, Madrid, Spain; 2 Hospital Universitario Fundación Jiménez Díaz, Instituto de Investigación Sanitaria Fundación Jiménez Díaz (IIS-FJD), UAM, Madrid, Spain; 3 Institut Catala d’Oncologia, Hospital Germans Trias i Pujol, Josep Carreras Leukemia Research Institute, Universitat, Autònoma de Barcelona, Badalona, Spain; 4 Institut Catala d’Oncologia – L’Hospitalet de Llobregat, L’Hospitalet de Llobregat, Spain; 5 Hospital Quiron Zaragoza, Zaragoza, Spain; 6 Hematology, Institut Catala d’Oncologia, Girona, Spain; 7 Hematology Department, Hospital Clínic, Barcelona, Spain; 8 Hematology Department, IBSAL-Hospital Universitario de Salamanca, CIC and CIBERONC, University of Salamanca, Salamanca, Spain; 9 Hematology Department, Hospital de Jerez de la Frontera, Jerez, Spain; 10 Hematology Department, Hospital Universitario y Politécnico La Fe, Valencia, Spain; 11 Hospital Mutua Terrassa, Terrassa, Spain; 12 Hematology, Hospital Virgen de la Victoria, Malaga, Spain; 13 Hospital U. Marqués de Valdecilla, Servicio de Hematología-Hemoterapia, Santander, Spain; 14 Hematology, Hospital Universitario de Burgos, Burgos, Spain; 15 Hospital General de Granollers, Barcelona, Spain; 16 Hospital Clínico Universitario de Santiago de Compostela, Santiago de Compostela, Spain; 17 Hospital Virgen de la Salud, Toledo, Madrid, Spain; 18 Hospital Doctor José Molina Orosa de Lanzarote, Arrecife, Spain; 19 Hospital General de Castellón, Castellón, Spain; 20 Hospital Universitario La Princesa, Madrid, Spain; 21 Hospital General de Alicante, Alicante, Spain; 22 Department of Hematology, Hospital Universitario de Canarias, La Laguna, La Laguna, Spain; 23 Hospital Universitario Puerto Real, Puerto Real, Spain; 24 Hematology Department, Hospital Universitario La Paz-Idipaz, Madrid, Spain; 25 Hospital de Jerez de la Frontera, Jerez de la Frontera, Spain; 26 Servicio de Hematología, Hospital Universitario Clínico Virgen de la Arrixaca, Murcia, Spain; 27 Instituto Ramón y Cajal de Investigación Sanitaria (IRYCIS), Hospital Universitario Ramón y Cajal, Madrid, Spain; 28 Hospital Regional Universitario de Málaga, Málaga, Spain; 29 Hospital Clinico de Valencia, Valencia, Spain

**Keywords:** Molecularly targeted therapy, Drug development

Dear Editor,

Despite the excellent overall survival (OS) of chronic myeloid leukemia (CML) patients, a significant proportion will fail currently available tyrosine-kinase inhibitors (TKIs) due to resistance or intolerance^1–3^. Intolerant patients are usually managed successfully with alternative second-generation tyrosine-kinase inhibitors (2GTKIs). However, more than half of the patients will eventually discontinue second-line treatment due to loss of response or toxicity^4,5^. Ponatinib is an effective drug in the setting of resistance to 2GTKIs, however with life-threatening side effects and varying responses^6–8^.

Asciminib is a first-in-class STAMP (Specifically Targeting the ABL Myristoyl Pocket) inhibitor that potently and specifically inhibits BCR-ABL1 via binding to a pocket distinct from the ATP binding site of the kinase^9^. Asciminib has the potential to overcome resistance to prior TKIs, and also offers the possibility of dual inhibition of BCR-ABL1 in combination with ATP-binding TKIs^10,11^. Asciminib has been evaluated in a phase I study in patients with Ph-positive leukemia failing prior TKIs, with promising results^12^.

Our aim is to share the first data on the use of asciminib in CML patients in clinical practice, allowed by Novartis under a managed-access program (MAP).

Data from 31 *BCR-ABL1*-positive CML patients treated with asciminib (median dose 40 mg BID) between October 2018 and June 2020 in 25 institutions from the Spanish CML Group (GELMC) were retrospectively collected.

Monitoring and response milestones followed the European Leukemia Net 2020 recommendations^13^. All patients were treated under the MAP by Novartis. MAP requests were independently reviewed by a licensed treating physician to confirm that the following criteria were met: (a) treatment need of a serious or life-threatening disease lacking commercially available options; (b) patient had to be ineligible or unable to participate in a clinical trial, and (c) the request should be in alignment with all applicable local laws and regulations. The study was approved by the Spanish Drug Agency and the Ethics Committee of the Hospital Universitario Ramón y Cajal (Madrid), with consent obtained from all the subjects. BCR-ABL1 analysis was not centralized but all samples were analyzed in EUTOS accredited laboratories.

Treatment emergent adverse events (TEAEs) were graded according to the National Cancer Institute Common Terminology Criteria for Adverse Events Version 4.0. Failure-free survival (FFS) is defined as time from first dose of asciminib to on-treatment death, progression to advanced phase, confirmed loss of complete cytogenetic response (CCyR), loss of complete hematologic response (CHR), treatment discontinuation for any reason (intolerance or lack of efficacy), or death for any reason. Progression-free survival (PFS) is defined as time from first dose of asciminib to on-treatment death, progression to advanced phase, loss of CHR, or death for any reason. Variables studied to identify factors associated with response to asciminib included resistance versus intolerance, resistance to previous TKIs, previous use of ponatinib, prior CCyR status, and presence of *BCR-ABL1* mutations.

Median time on previous TKI treatment until asciminib initiation was 77 months (range 6–221 months). Patients’ baseline characteristics are compiled in Table 1. Patients were heavily pretreated with 28 of them having received 3 or more TKIs previous to asciminib. Eleven patients (35%) had been treated with ponatinib. Twelve patients (39%) had baseline BCR*-ABL1* mutations (1 case with T315I mutation). Switch to asciminib was due to intolerance to prior TKIs in 22 patients and due to resistance in the remaining 9. Starting asciminib dose was 40 mg BID in all patients except in the single case harboring the T315I mutation, who received 200 mg BID.

**Table 1:** Patients baseline characteristics.

Patients’ characteristics (*n* = 31)	
Age at data collection, yr (range)	69 (22–89)
Age at diagnosis, yr	55
Female sex % (*n*)	65 (20)
Time on previous TKIs, months (range)	77 (13–221)
Disease stage before asciminib % (*n*)	
Chronic phase	96 (30)
Accelerated phase	3 (1)
Blast phase	0
Sokal risk % (*n*)	
Low	32 (10)
Intermediate	36 (11)
High	33 (10)
TKI at diagnosis % (*n*)	
Imatinib	74 (23)
Dasatinib	10 (3)
Nilotinib	13 (4)
Bosutinib	3 (1)
Ponatinib	0 (0)
≥3 prior TKI lines % (*n*)	90 (28)
Prior use of ponatinib % (*n*)	35 (11)
BCR-ABL1 mutations % (*n*)	38 (12)
E255K % (*n*)	25 (3)
Exon 7 % (*n*)	17 (2)
T315I % (*n*)	8 (1)
Others % (*n*)	50 (6)

Median duration on asciminib treatment at time of analysis was 8.8 months (8.2 and 9.3 in resistant and intolerant patients, respectively). Median follow-up for the entire population was 10.2 months.

Overall, 58% of patients had ≥1 TEAE of any grade and 32% had ≥1 TEA of grade 3-4. Thirteen patients (42%) experienced mild non-hematological side effects (grade 1-2), the most frequent being fatigue (19%), joint pain (16%), and nausea (9%). Four patients (12%) had severe (grade 3-4) non-hematological events: fatigue, hepatotoxicity, hypertension, and pericardial effusion (1 patient each). Three patients (9.7%) developed grade 4 thrombocytopenia (all of them had previously experienced same toxicity with previous TKIs), 2 of them combined with grade 4 neutropenia. One of the 4 patients who had suffered from pancreatitis on prior TKI treatment presented the same AE under asciminib, which resolved following treatment discontinuation. The patient receiving 200 mg BID due to T315I mutation suffered a total of three grade 1 AEs: thrombocytopenia, fatigue, and abdominal pain.

Dose reduction (median reduced dose was 40 mg once a day), mostly due to hematological toxicity, was required in 9 patients (29%), with temporary treatment interruption in 7 cases. Probabilities of suffering grade 3-4 TEAE were 44% and 22% for resistant and intolerant patients, respectively.

Four patients discontinued treatment due to progression to blast phase (*n* = 2) and lack of efficacy (*n* = 2). No patients discontinued treatment due to TEAEs. 2 patients died during the observation period due to progression. At last evaluation, 27 patients (87%) remained on asciminib treatment.

At the time of analysis, with a median time on asciminib of 8.8 months, the cumulative responses rates (including reaching or at least maintaining previous response) of CHR, CCyR and major molecular response (MMR) were 100%, 66%, and 41%, respectively. A total of 17 patients (55%) and 28 patients (90%) improved or maintained baseline response, respectively during follow-up (Table 2).

**Table 2:** Response to asciminib subanalysis regarding baseline response.

	Resistant (9)	Intolerant (19*)	Total (29*)
Best response to asciminib			
All patients			
CHR^a^	9/9 (100%)	19/19 (100%)	29/29 (100%)
CCyR^a^	3/9 (33%)	16/19 (84%)	19/29 (66%)
MMR^a^	1/9 (11%)	11/19 (58%)	12/29 (41%)
MR4.5^a^	0/9 (0%)	4/19 (21%)	4/29 (14%)
Patients without response at baseline			
CCyR^b^	3/9 (33%)	5/8 (63%)	8/17 (47%)
MMR^b^	1/9 (11%)	7/15 (47%)	8/24 (33%)
MR4.5^b^	0/3 (0%)	4/19 (21%)	4/29 (14%)

*Due to short follow-up, 2 patients were excluded from response analysis.

*CHR* complete hematological response, *CCyR* complete cytogenetic response, *MMR* major molecular response, *MR4.5* detectable disease with BCR-ABL1IS < 0.0032%.

^a^Patients with CHR, CCyR, MMR, or MR4.5 at baseline were evaluable for hematologic, cytogenetic, or molecular response and were considered responders if they maintained their response. ^b^Evaluable patients without a CCyR, MMR, or MR4.5 at baseline.

In patients without the respective responses at baseline, cumulative rates of CCyR and MMR were 48% (8/17) and 33% (8/24), respectively. Probabilities to obtain CCyR and MMR in resistant and intolerant patients were 33% (3/9) vs 62% (5/8) and 11% (1/9) vs 47% (7/15), respectively.

Amid the 11 patients previously treated with ponatinib, 3 patients (27%) showed improvement of response achieving at least MMR. Two were in TKI-intolerant group and one in the TKI-resistant group.

Regarding the 12 patients with *BCR-ABL1* baseline mutations, 4 patients (44%) improved baseline response: 2 patients (carrying M344I and E255V mutations reached CCyR and 2 patients improved their responses to MMR (both carrying exon 7 deletion). The patient with T315I mutation suffered eventually from CHR loss after 3 months and asciminib was interrupted due to progression. With a median follow-up of 10.2 months, the estimated FFS, PFS, and OS were 87%, 94%, and 94%, respectively.

We analyzed the factors associated with the response to asciminib, identifying the achievement of CCyR with prior TKI treatment as the only predictive factor (OR 10.5; 95% CI: 1.02–108.6).

To our knowledge, the present study is the first to describe the results of asciminib treatment in CML patients managed in clinical practice under a compassionate access program. It is important to highlight the differences between our study population and that of the previously published paper from the phase I clinical trials with asciminib^12^. In the phase I trial, patients could receive asciminib after failing at least two TKIs, while in the present study patients did not have any alternative effective treatment. Taking into consideration that the majority of patients in our study were classified as intolerant to previous TKIs, the population should be considered as at special high risk of suffering side effects. Our data confirmed the good safety profile of asciminib in a heavily treated real-world population. No patients discontinued asciminib due to side effects, which is in line with previous asciminib studies (with shorter follow-up) that showed probabilities of treatment discontinuations due to side effects to be less than 10%^11^. This information contrast with data from 2GTKI studies in which probabilities to discontinue treatment due to side effects are higher^6^. Besides good data regarding asciminib tolerability, our study suggests how patients that suffer from pancreatitis or hematological toxicities with previous TKIs are at risk of suffering same side effects with asciminib.

Regarding efficacy, overall responses to asciminib were similar to the ones in phase 1 trial, with 61% of patients achieving or maintaining CCyR. Despite adequate overall responses, it is important to mention that probabilities to achieve CCyR in resistant patients without CCyR at baseline was 33% (3/9), which is significantly lower compared to results observed in phase I trial. One explanation could be the more pretreated population in our study; however, ongoing studies will clarify probabilities of achieving optimal responses in patients without baseline responses.

Our study included 30% of patients that had received ponatinib prior to asciminib. As expected, only 3/9 patients show improved responses, with 2 of these patients corresponding to the intolerance group. The phase I study also included 30% of patients previously treated with ponatinib, nevertheless, treatment responses according to previous ponatinib exposure were not reported to date. We believe that further asciminib studies should describe asciminib activity in ponatinib-resistant patients in order to clarify best treatment alternatives in 2GTKI-resistant patients^14^.

In conclusion, the results presented, in line with data from the phase 1 study, show asciminib as a safe and efficacious drug for CML patients without treatment alternatives in common clinical practice. Further ongoing studies will provide information related the type of patients that would benefit the most from this new treatment approach.
